# Thermal inactivation kinetics and effects of drying methods on the phenolic profile and antioxidant activities of chicory (*Cichorium intybus* L.) leaves

**DOI:** 10.1038/s41598-018-27874-4

**Published:** 2018-06-22

**Authors:** Ran Li, Hongmei Shang, Hongxin Wu, Menghan Wang, Mengying Duan, Junyan Yang

**Affiliations:** 1 College of Animal Science and Technology, Jilin Agricultural University, Changchun, 130118 China; 2 Key Laboratory of Animal Nutrition and Feed Science of Jilin Province, Changchun, 130118 China; 3 Key Laboratory of Animal Production, Product Quality and Security, Ministry of Education, Changchun, 130118 China; 4 Grassland Research Institute of CAAS, Hohhot, 010010 China

## Abstract

The thermal inactivation kinetics of enzymes, including polyphenol oxidase (PPO) and peroxidase (POD), in chicory (*Cichorium intybus* L.) leaves were evaluated. In addition, the influences of different drying techniques (shade drying, hot air drying and freeze drying) on the phenolic profiles and antioxidant activities of chicory leaves were determined. The antioxidant activities of chicory leaves were evaluated on the basis of their 1,1-diphenyl-2-picrylhydrazyl (DPPH) radical scavenging activity, reducing power, and 2,2-azino-bis-(3-ethylbenzothiazoline-6-sulfonic acid) (ABTS) radical scavenging activity. The results showed that the activation energy for PPO and POD inactivation were 123.00 kJ/mol and 78.99 kJ/mol, respectively. Preliminary treatment with hot water for 3 min at 90 °C was beneficial for preserving the phenolics present in fresh leaves. Hot air drying was better for the phenolics preservation. The hot air-dried and freeze-dried leaves possessed good antioxidant activities. The leaves with higher phenolics contents had better antioxidant activities, which indicated that the preservation of the phenolics was important for maintaining the antioxidant activity of chicory leaves.

## Introduction

Chicory (*Chicorium intybus* L.), a major crop in northwestern Europe, has been used in indigenous medicine for hundreds of years^1,2^. In fact, chicory cultivation has multiple purposes; its leaves can be used as a vegetable and forage crop, and its roots can be used to produce both inulin and a coffee substitute^3^. Chicory has potent hepatoprotective, antioxidant, hypoglycemic, hydragogue and immunoregulatory activities^4^. It has been reported that chicory leaves contain a high content of phenolics (190 ± 2.03 mg/g of the dry matter)^5^. The content of phenolic compounds is strongly correlated with antioxidant capacity in plant samples, which suggests that chicory leaves are a good source of antioxidants^6^. However, the shelf-life of fresh chicory leaves is short because of enzymatic reactions and microbial growth. Thermal treatment, a preliminary processing method, and drying, a preservation technique, are deemed to be the most effective methods for preserving the quality of fresh materials^7^.

Polyphenol oxidase (PPO) is widespread in plant materials. The chemical conversion of phenolic compounds to quinones is catalyzed by PPO and leads to enzymatic browning and the loss of phenolics in fresh plant materials^8^. Peroxidase (POD) is also rich in plant materials. The chemical oxidation of phenolic compounds to phenoxy radicals is catalyzed by POD with hydrogen peroxide and causes the oxidation of chlorophyll^9^. Because of its thermostability and high content in most plants, POD is often used as an indicator in thermal food processing treatments^10^. Hot water blanching, an important thermal treatment method, is normally used for the inactivation of enzymes. The differences between the parameters used for heat-labile and heat-resistant isoenzyme fractions suggest the importance of the kinetics of POD and PPO inactivation in different raw materials^7^. However, no comparative studies have been reported on the enzyme inactivation kinetics of chicory leaves, which is important for the retention of their initial quality.

In addition, drying methods play an important role in food processing, and it has two vital functions: inhibiting microbial growth and facilitating storage^11^. To some extent, drying can affect the initial quality in terms of appearance and the preservation of unstable components. To date, several drying methods have been used to dehydrate plant materials^7^. Sun and shade drying are two common drying methods for fresh plant materials. They are operationally simple and are inexpensive. Shade drying is beneficial for the preservation of sun-unstable components. Hot air drying is applied to accelerate the drying process. However, oxidation or pyrolysis reactions during sun drying, shade drying or hot air drying may affect the chemical components of the plant materials^12^. Because of the low-temperature and low-pressure environment, freeze drying has advantages for preserving the quality of plant materials^13^. However, although freeze drying is beneficial for the preservation of sensory attributes, it might cause the loss of active ingredients^14^. Different drying methods affect the characteristic chemical compounds and the antioxidant activities of medicinal plants in different ways^11^. The effect of a particular drying method on the retention of raw quality is not predictable and depends on the compounds and the specific plant involved. Therefore, a significant amount of information for improving the qualities of products such as functional food ingredients or nutraceuticals can be revealed by the comparative evaluation of various drying technologies. However, information on the changes in the phenolic profiles and antioxidant activities of chicory leaves after drying with different methods is very limited.

Propper postharvest treatments (including preliminary processing and preservation technique) of chicory leaves possessing high levels of phenolics and antioxidant activities are essential for the retention of their initial quality. Therefore, the purposes of this research were: (1) establishing the mathematic relation between hot water blanching and enzyme (PPO and POD) inactivation by an enzymatic kinetic model and (2) evaluating the phenolic profiles and antioxidant activities of chicory leaves after drying by different techniques (shade, hot air and freeze drying).

## Results and Discussion

### Kinetic parameters of PPO and POD inactivation during thermal inactivation

**Figure 1:**
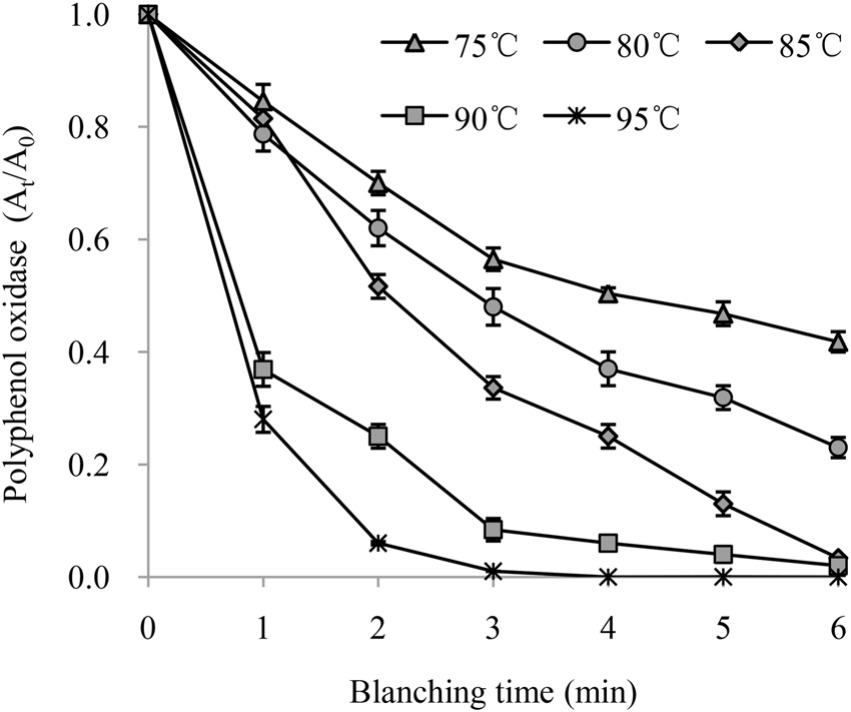
Residual polyphenol oxidase activity in chicory leaves during water blanching.

The kinetic parameters for PPO and POD inactivation after water blanching are shown in Table 1. The changes in the residual PPO activity versus water blanching time at different temperatures (75, 80, 85, 90 and 95 °C) are shown in Fig. 1. The PPO activity in chicory leaves was significantly influenced by the blanching temperature and duration. Due to the high determination coefficients (*R*^2^) in the 0.9398 to 0.9974 range, the experimental results were well fit by a first-order kinetic model of enzymatic reactions under these experimental conditions. The *k* values of PPO inactivation in chicory leaves increased from 0.158 to 1.751 as the blanching temperature increased. The activation energy for PPO inactivation was 123.00 kJ/mol based on the calculation shown in Fig. 1.

**Table 1:** Kinetic parameters of the first-order kinetic model for PPO and POD inactivation by water blanching.

Temperature (°C)	PPO		POD	
	*k*/min	*R* ^2^	*k*/min	*R* ^2^
75	0.158	0.9886	0.273	0.9858
80	0.238	0.9974	0.468	0.9851
85	0.464	0.9756	0.663	0.9778
90	0.633	0.9905	1.097	0.9758
95	1.751	0.9398	1.133	0.9666

PPO, polyphenol oxidase; POD, peroxidase.

**Figure 2:**
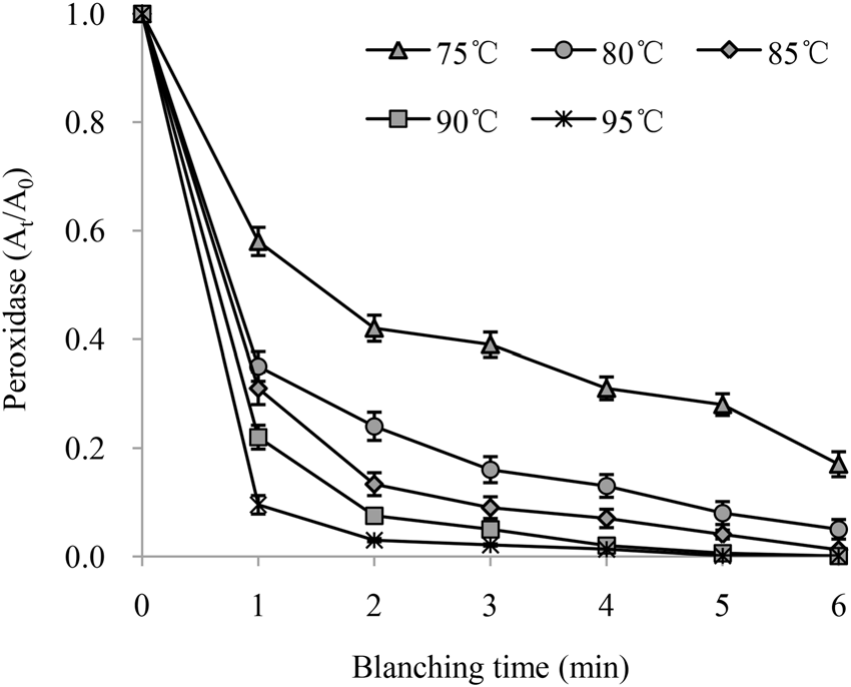
Residual peroxidase activity in chicory leaves during water blanching.

The plots of residual POD activity in the chicory leaves versus blanching time at five different temperatures (75, 80, 85, 90 and 95 °C) are shown in Fig. 2. Similar to PPO, the water blanching temperature and duration had significant influences on the POD activity in chicory leaves. According to the high determination coefficients (*R*^2^), which ranged from 0.9666 to 0.9858, the experimental results were well fit by a first-order kinetic model of enzymatic reactions under the tested temperatures. As the blanching temperature increased, the *k* values of POD inactivation in chicory leaves ranged from 0.273 to 1.133. The activation energy for POD inactivation was 78.99 kJ/mol based on the analysis shown in Fig. 2. The activation energy of PPO inactivation was higher than that of POD, which indicated PPO was more heat resistant than POD, and PPO was recommended as the enzymatic reference material for the heat treatment of chicory leaves.

### Amount of total phenolics preserved and DPPH radical scavenging activity after thermal inactivation

Hot water blanching is a common method of inactivating enzymes and preserving the initial quality of fresh materials. However, some thermally sensitive compounds including phenolics may lose their activities due to oxidization or diffusion into the water during hot water treatments. Nevertheless, the preservation of phenolics is important for maintaining the biological activities of the samples^5^. Therefore, the amount of phenolic compounds remaining after blanching can be used as an indicator for estimating the quality of chicory leaves.

**Figure 3:**
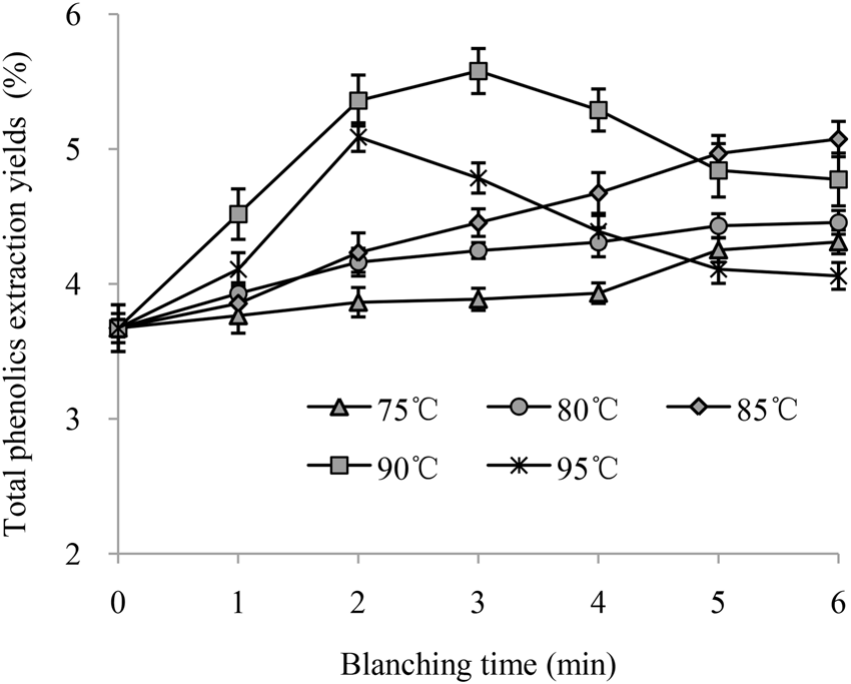
Total phenolics extraction yields of chicory leaves after water blanching.

**Figure 4:**
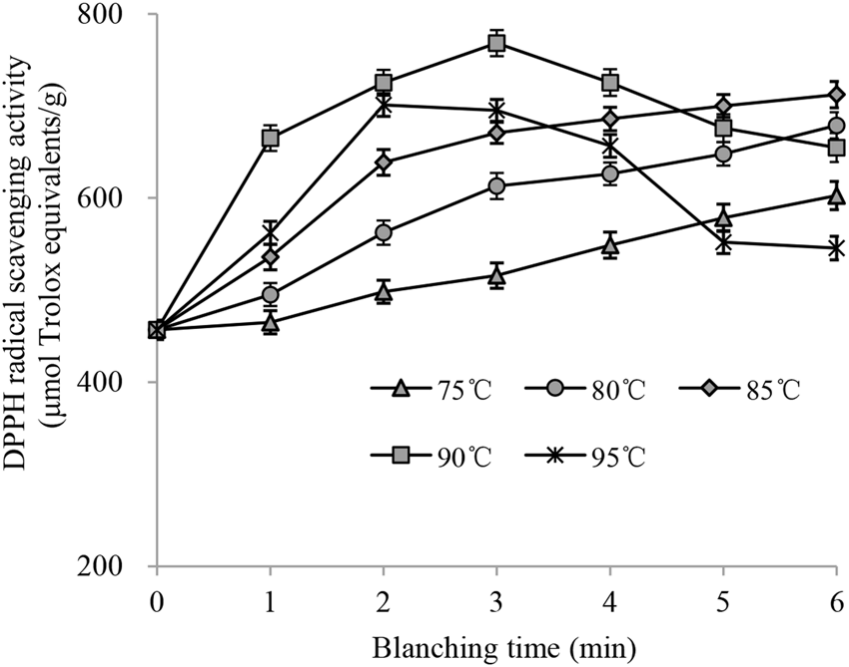
DPPH radical scavenging activity of chicory leaves after water blanching.

The total phenolics extraction yields of chicory leaves after water blanching are shown in Fig. 3. At temperatures of 75, 80 and 85 °C, the extraction yields of total phenolics improved as the blanching time increased. These results may be due to the increased thermal inactivation of PPO and POD in chicory leaves as the treatment time increased at 75, 80 and 85 °C. The yield of total phenolics extracted from the leaves which were subjected a preliminary water blanching treatment for 3 min at 90 °C (5.58 ± 0.17%) was higher than those of other blanching temperatures. One possible reason was that treatment at higher temperatures may more effectively inactivate the PPO and POD in chicory leaves. Nevertheless, the total phenolics extraction yields decreased as the blanching time exceeding 2 min at 95 °C or 3 min at 90 °C (*P* < 0.05). This may be due to the serious thermal degradation of phenolics that occurs at 90 and 95 °C when blanching for an extended period. In addition, the phenolics extraction yields of leaves treated with hot water for 5–6 min at 95 °C were lower than those of leaves treated for the same time at 75, 80, 85 and 90 °C. Lin *et al*. found that the phenolics yields from *Rabdosia serra* (Maxim.) Hara leaf decreased as the blanching temperature increased from 70 to 100 °C, and the total phenolics yields decreased as the blanching time exceeding 4 min at 70 and 100 °C or 5 min at 90 °C^7^. As shown in Fig. 4, the DPPH radical scavenging activities of chicory leaves followed the same trends when blanched at different temperatures for 1 to 6 min, which indicated that the preservation of the phenolics is important for preserving the DPPH radical scavenging activity of chicory leaves.

Therefore, considering the effectiveness of enzymatic inactivation and the preservation of total phenolics and the DPPH radical scavenging activity, the chicory leaves should be blanched with hot water for a relatively short time at a higher temperature. Water blanching for 3 min at 90 °C is recommended as the most effective thermal treatment for chicory leaves.

### Assessment of different drying methods for chicory leaves

The moisture contents of leaves after dehydration using different methods decreased in the following order: shade-dried leaves (12.54 ± 0.18%) > freeze-dried leaves (12.07 ± 0.31%) > hot air-dried leaves (11.66 ± 0.12%) (*P* > 0.05). During freeze drying, the moisture in the plant materials was first frozen and then lost through sublimation. During shade drying and hot air drying, moisture is normally lost through evaporation. The key difference between these methods is that hot air could improve the water loss efficiency during hot air drying.

### Inactivation kinetic parameters of PPO during drying processes

**Figure 5:**
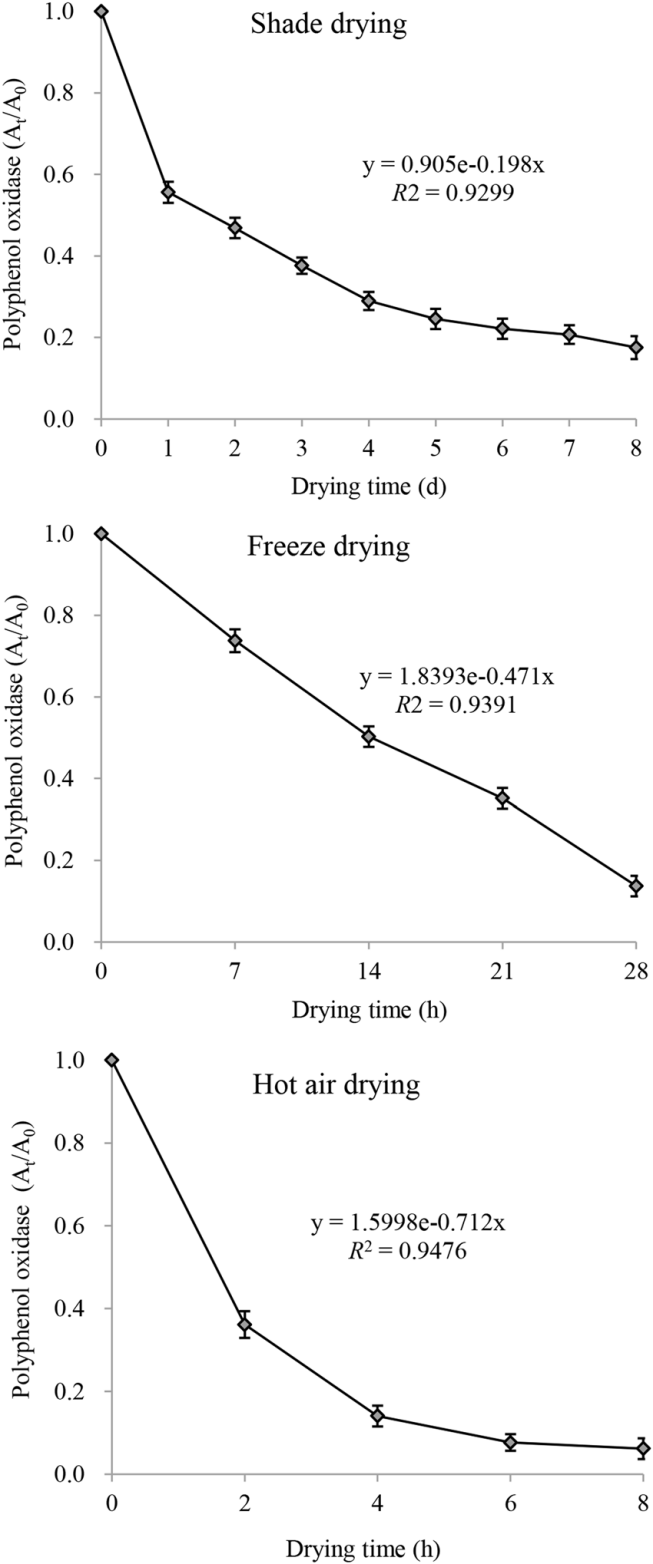
The kinetic parameters and residual activities of polyphenol oxidase in chicory leaves during different drying processes.

Based on the experiments on the thermal inactivation of enzymes conducted in this study, PPO is recommended as the enzyme reference material for the inactivation treatments of chicory leaves. Therefore, the PPO activity in chicory leaves was measured during different drying processes. The kinetic parameters and residual activities of PPO in chicory leaves during different drying processes are shown in Fig. 5. Due to the high determination coefficients (*R*^2^), which were in the 0.9299 to 0.9476 range, the experimental results were well fit by a first-order kinetic model of enzymatic reactions under the experimental conditions for these drying methods. The residual activities of PPO at the end of shade drying and freeze drying were 0.175 and 0.137, respectively. The residual activity of PPO was 0.062 after hot air drying. Therefore, hot air drying was more effective for PPO inactivation than shade drying and freeze drying.

### Phenolic compounds of chicory leaves dried by different methods

Different drying techniques had significantly effects on the total phenolics extraction yields of chicory leaves (*P* < 0.05). The total phenolics yields of leaves after drying with the different methods decreased in the following order: hot air-dried leaves (2.68 ± 0.09%) > freeze-dried leaves (2.34 ± 0.06%) > shade-dried leaves (1.35 ± 0.06%). Moreover, the influence of the drying method on the phenolic profile (chicoric acid, ferulic acid, chlorogenic acid, and caffeic acid) of chicory leaves was measured (Table 2). The content of chlorogenic acid in chicory leaves was highest among the tested phenolic compounds, followed by the contents of caffeic acid, ferulic acid and chicoric acid. The contents of chlorogenic acid and caffeic acid were significantly different among leaves dried by the three different methods (*P* < 0.05). The chlorogenic acid and caffeic acid contents in the leaves decreased in the following order: hot air-dried leaves > freeze-dried leaves > shade-dried leaves. The ferulic acid and chicoric acid contents in the leaves dried with hot air were also higher than those of leaves dried with the other two methods (*P* < 0.05). There were no significant differences in the contents of chicoric acid and ferulic acid between the shade-dried and freeze-dried leaves (*P* > 0.05). Hot air drying is recommended as a beneficial drying method for chicory leaves for preserving the phenolics constituents based on the current study.

**Table 2:** Phenolic profile of chicory leaves after different drying methods (mg/g).

Items	Shade drying	Freeze drying	Hot air drying
Chlorogenic acid	1.756 ± 0.117^c^	2.432 ± 0.186^b^	4.558 ± 0.173^a^
Caffeic acid	0.510 ± 0.009^c^	0.580 ± 0.003^b^	0.644 ± 0.032^a^
Chicoric acid	0.033 ± 0.004^b^	0.020 ± 0.005^b^	0.137 ± 0.023^a^
Ferulic acid	0.022 ± 0.001^b^	0.036 ± 0.003^b^	0.350 ± 0.043^a^

Means in the same row (same phenolic compound) with different superscripts (a–c) differ significantly (P < 0.05). Data are the means ± SD (n = 3).

Hot air drying is one of the most commonly used dried methods in food industry and is inexpensive. Shade and freeze drying of chicory leaves caused losses of phenolics in this study. Losses of phenolics during drying can mainly be attributed to oxidative reactions. During shade drying and hot air drying, both non-enzymatic and enzymatic oxidative reactions are likely taking place in the plant material. However, hot air drying can inhibit enzymatic oxidative reactions to some extent due to the inactivation of enzymes at 60 °C (Fig. 5). Enzymatic oxidation by PPO and POD is more likely to occur during freeze drying due to the lower exposure to oxygen and to the damage to the cell structure caused by ice crystals formation^12^. Therefore, freeze drying caused a greater loss in the contents of total phenolics, chlorogenic acid, caffeic acid, ferulic acid and chicoric acid than hot air drying (Table 2). These differences can be explained by the formation of ice crystals within the tissue matrix during freeze drying, leading to cell rupture and exposure of the phenolic species to the aforementioned oxidative conditions^15^. Freeze drying of guava powders and pumpkin also led to the loss of greater contents of phenolic compounds than hot air drying^12,14^. However, in most cases, freeze dried products exhibit much higher contents of bioactive compounds than hot air dried products. Some studies have shown that the freeze drying of grape skin, tomatoes, ginger and citrus fruits leads to higher contents of phenolic compounds than hot air drying^16–18^. Therefore, whether hot air or freeze drying will be better for preserving the content of phenolic compounds in a given fruit or vegetable is difficult to predict based on data from other foods and needs to be supported by experimental data from the relevant plant.

### Antioxidant activities of chicory leaves dried by different methods

The DPPH free radical is a kind of stable free radical. The ability to scavenge the DPPH radical is widely used to estimate the antioxidant activities of biological samples^19^. The antioxidant substances can reduce the DPPH radical to diphenyl-picrylhydrazine, which is yellow^20^. The antioxidant ability of a sample is highly correlated with the content of the yellow compound in the reaction system. The ABTS free radical is also stable, and the ABTS radical scavenging assay only needs a short reaction time (approximately 15 min), and this method has been widely used to assess the antioxidant capacities of various samples^21^. There is a positive correlation between the antioxidant capacity and the reducing power of a sample^22^. The free radical chain can be broken down by an antioxidant by providing a hydrogen atom or by the reaction with a peroxide precursor, which concomitantly inhibits the formation of peroxide^23^.

The DPPH radical scavenging activities, reducing powers, and ABTS radical scavenging activities of chicory leaves dehydrated by different methods are present in Table 3. Higher numerical values represent stronger antioxidant capacities. The leaves dried with hot air possessed stronger ferric reducing power and DPPH radical scavenging activity than leaves dehydrated by freeze drying or shade drying (*P* < 0.05). The ABTS radical scavenging activities of the hot air-dried and freeze-dried leaves were not significantly different (*P* > 0.05). The leaves dehydrated by shade drying exhibited lower antioxidant activities than the leaves dried by hot air or freeze drying (*P* < 0.05).

**Table 3:** Antioxidant activities of chicory leaves after different drying methods (μmol Trolox equivalents/g).

Items	Shade drying	Freeze drying	Hot air drying
DPPH radical scavenging activity	165.0 ± 10.1^c^	346.4 ± 12.5^b^	389.7 ± 9.90^a^
ABTS radical scavenging activity	139.2 ± 8.20^b^	264.7 ± 14.3^a^	280.3 ± 11.1^a^
Ferric reducing power	232.2 ± 13.7^c^	430.3 ± 13.6^b^	458.2 ± 13.1^a^

Means in the same row (same antioxidant assay) with different superscripts (a–c) differ significantly (*P* <0.05). Data are the means ± SD (n = 3).

The chicory leaves with higher total phenolics contents possessed greater antioxidant capacities. Correlation analysis indicated that the total phenolics contents of the leaves was positively correlated with their DPPH radical scavenging activity (*r* = 0.998, *P* < 0.05). These results showed that the preservation of phenolics was important for the retention of the antioxidant activity of chicory leaves.

In conclusion, severe enzymatic degradation can be catalyzed by PPO and POD in harvested fresh chicory leaves. For enzymatic inactivation, PPO is recommended as the reference enzyme for the heat treatments of chicory leaves because PPO has a higher activation energy than POD. Preliminary treatment with hot water for 3 min at 90 °C was beneficial for the retention of the phenolic compounds in fresh leaves. The phenolic profiles and antioxidant activities of chicory leaves were significantly influenced by the drying techniques. Hot air drying was better for the preservation of phenolic compounds in chicory leaves. The hot air-dried and freeze-dried leaves possessed good antioxidant activities. The leaves with higher phenolics contents had stronger antioxidant activities, which indicated that phenolics preservation is important to maintain the antioxidant activity of chicory leaves.

## Materials and Methods

### Chicory leaves and reagents

Fresh aboveground parts of chicory were harvested in Jilin Agricultural University (Jilin, China). The plants were in the bloom stage. Leaves were separated from the stems and cleaned with water without damaging the leaves. All the chemicals used in high-performance liquid chromatography (HPLC) were of HPLC-grade. DPPH, gallic acid, Folin-Ciocalteu reagent, ABTS, chicoric acid, ferulic acid, caffeic acid, and chlorogenic acid were bought from Sigma-Aldrich (St. Louis, USA). All other reagents were of analytical-grade and were obtained from local suppliers.

### Thermal inactivation treatments

For the heat treatment of chicory leaves with hot water, 50 g of fresh leaves was treated at 75, 80, 85, 90 and 95 °C for 1 to 6 min. After treatment in a hot water bath, the leaves were quickly cooled with ice water to terminate the thermal inactivation. A twenty-gram sample of fresh or blanched leaves was homogenized with a lab blender (JJ-2, Changzhou Lang Yue Instrument Manufacturing Co., Ltd., Changzhou, China) with 200 mL of 95% (v/v) ethanol. The homogenate was subjected to ultrasonic treatment using an ultrasonic processer (KQ-100KDE, Kunshan ultrasonic instruments Co., Ltd., Kunshan, China) at room temperature for 60 min to extract the total phenolics. Next, centrifugation (3K30, Sigma, Germany) at 4 °C and 3000 *g* for 10 min was used to separate the supernatant from the extract homogenate. The supernatant was adjusted to a volume of 200 mL by 95% (v/v) ethanol and prepared for the determination of total phenolics content and DPPH radical scavenging activity following thermal inactivation treatment. The DPPH radical scavenging activity is reported in micromole Trolox per gram of chicory leaves (dry basis). The total phenolics extraction yield was calculated by using the following equation (1):

1 $${\rm{Total}}\,{\rm{phenolics}}\,{\rm{yield}}\,( \% )=\frac{C\times V}{W}\times 100$$

where *C* is the total phenolics concentration by mass in the chicory leaf extract, *V* is the volume of chicory leaf extract, and *W* is the weight of chicory leaves (dry basis) used to prepare the chicory leaf extract.

### Measurement of PPO and POD activities

The enzymes in the blanched chicory leaves were extracted and purified based on the method reported by Ünal and Sener^24^. The extraction and purification processes were carried out at 4 °C. The crude extract was purified by DEAE-Toyopearl 650 M and Sephadex G-100 gel column chromatography. The PPO and POD activities and protein content were monitored in the collected fractions (3 mL). The fractions showing maximum PPO activity and POD activity were separately pooled and used to analyze the enzyme inactivation kinetics of PPO and POD.

The method used for measuring the PPO activity was adapted from Augusto *et al*.^25^ using pyrogallol as the substrate^25^. Pyrogallol (40 mM) was dissolved in phosphate buffer (0.2 M, pH 7). The reaction system was composed of enzyme extract (0.25 mL) and pyrogallol solution (2.75 mL). The blank was the same mixture except the enzyme extract was replaced with the same volume of phosphate buffer (0.2 M, pH 7). The absorbance was recorded at a wavelength of 420 nm.

The method used for measuring the POD activity was adapted from Tan *et al*.^26^. Guaiacol was used as the substrate. The reaction system was composed of phosphate buffer (0.2 M, 1.7 mL, pH 6), guaiacol (40 mM, 1 mL), hydrogen peroxide (40 mM, 1 mL), and the enzyme extract (0.3 mL). The blank was composed of the same reagents except additional phosphate buffer (0.2 M, 0.3 mL, pH 6) was used instead of the enzyme extract. The absorbance was measured at 470 nm.

To determine the enzymes activities of PPO and POD, absorbance values were read every 20 seconds for 30 min. A curve was established using time as the abscissa and absorbance as the ordinate. The rate of the enzymatic reaction was calculated from the linear portion of the curve. The amount of enzyme that induced a 0.01 increase in the absorbance every minute was defined as one unit of PPO or POD activity^27^.

### Enzyme inactivation kinetic models

It was reported that a first-order inactivation kinetic model was suitable for describing the enzyme inactivation of PPO and POD during hot water treatment of the plant material^28^. The kinetic parameters of PPO and POD inactivation were calculated according to the following equation (2):

2 $${A}_{t}/{A}_{{\rm{0}}}=\exp (-kt)$$

where *A*_t_ is the enzyme activity at time t; *A*_0_ is the enzyme activity at time = 0; *k* is the first-order rate constant; and *t* is the hot water treatment time.

In addition, the thermal treatment temperature and *k* are related by the Arrhenius equation as follows (3):

3 $$k={k}_{ref}\exp [-\frac{{E}_{a}}{R}(\frac{1}{T}-\frac{1}{{T}_{ref}})]$$

where *k*_*ref*_ is the rate constant of a reference temperature (*T*_*ref*_); *E*_*a*_ is the activation energy of the enzymatic reaction; *R* is the gas constant; and *T* is the absolute temperature.

### Drying methods of fresh leaves

Three dehydration methods (shade drying, hot air drying, and freeze drying) were used to dry the chicory leaves. Shade drying was done in a shady and drafty room (20 °C, relative humidity of 45%) for 8 days. Hot air drying was done in a drying oven at 60 °C for 8 h, and the air flow rate was 2.0 m·s^−1^. Freeze drying was done in a vacuum freeze dryer (SCIENTZ-12N, Ningbo Scientz Biotechnology Co., Ltd., Ningbo, China) at −70 °C for 28 h. The PPO activity of the chicory leaves was measured as described in the ‘Measurement of PPO and POD activities’ section above following the thermal inactivation treatments. The moisture contents of the chicory leaf samples were measured by oven drying at 105 °C.

### Extraction of phenolic compounds from chicory leaves after drying

The dried chicory leaves were ground into powders and passed through a 1 mm sieve. One gram of the dried chicory leaf powder was mixed with 95% aqueous ethanol (25 mL), and the mixture was subjected to ultrasonic treatment in an ultrasonic bath (KQ-100KDE, Kunshan ultrasonic instruments Co., Ltd., Kunshan, China) at room temperature for 60 min. After extraction, the samples were centrifuged for 10 min (4 °C, 3000 *g*). The supernatant was adjusted to 25 mL by aqueous ethanol (95%) and was prepared for the determination of the total phenolics content and antioxidant activities and HPLC analysis of phenolic compounds.

### Measurement of the total phenolics content

The Folin–Ciocalteu method was used to assay the total phenolics contents of the chicory leaf extracts^29,30^. Distilled water (6 mL) and Folin–Ciocalteu reagent (0.5 mL) were mixed with chicory leaf extract (0.1 mL). The mixture was kept at room temperature for 3 min. Aqueous sodium carbonate solution (20%, 1.5 mL) and distilled water (1.9 mL) were added into the mixture. The mixture was incubated at room temperature for 60 min in a dark place. The absorption was assayed at 760 nm against a blank. The blank consisted of the same mixture except the chicory leaf extract was replaced with 0.1 mL of aqueous ethanol (95%). The standard curve used for the calculation of the total phenolics content was calibrated with gallic acid.

### Analysis of the phenolic compounds

The method used to determine the phenolic compounds was adapted from Kaewnarin *et al*.^31^ and used an LC-2010ATH HPLC instrument (Shimadzu, Japan) coupled to a UV detector^31^. The chicory leaf extract was separated on an Amethyst C18-H column (250 × 4.6 mm, 5.0 μm). The mobile phase was composed of 0.1% aqueous phosphoric acid and 100% acetonitrile (20:80, v:v). The column oven temperature, detection wavelength, injection volume, and flow rate were 25 °C, 327 nm, 20 μL, and 1 mL/min, respectively. All the phenolic acids, including chicoric acid, caffeic acid, chlorogenic acid, and ferulic acid, were quantified using an external standard (Fig. S1).

### Antioxidant activities

The antioxidant activities of the chicory leaf extracts were evaluated using the DPPH radical scavenging activity^32^, ferric reducing power^33^, and ABTS radical scavenging activity assays^34^. The equivalent antioxidant activities were determined relative to a standard curve of Trolox, and the values are reported in micromole Trolox per gram of the chicory leaf sample (dry basis).

### Statistical analysis

All treatments were carried out in triplicate, and all sample analyses were performed three times and averaged. The results are reported as the mean ± standard deviation (SD). One-way ANOVA in SPSS 17.0 for Windows (SPSS Inc., Chicago, IL) was performed for statistical analysis. Differences at *P* < 0.05 were considered to be statistically significant by Duncan’s multiple-range test. Linear regressions were used to estimate the linear correlation between two sets of parameters. Regression parameters at 0.05 probability were regarded as a significant correlation.

### Data Availability

All data generated or analyzed during this study are included in this published article.

## Electronic supplementary material

Supplementary Information
