# Supplementary material for: Thermal inactivation kinetics and effects of drying methods on the phenolic profile and antioxidant activities of chicory (Cichorium intybus L.) leaves
Source: Sci Rep. 2018 Jun 22;8:9529. doi: 10.1038/s41598-018-27874-4 (PMC6015010; doi:10.1038/s41598-018-27874-4)
Supplement: Supplementary file 1 — Supplementary Information [file 41598_2018_27874_MOESM1_ESM.pdf]

Supplementary information for

Thermal inactivation kinetics and effects of drying methods on the phenolic profile and antioxidant activities of chicory (*Cichorium intybus* L.) leaves

Ran Li[1], Hongmei Shang[1,2,3*] , Hongxin Wu[4], Menghan Wang[1], Mengying Duan[1], Junyan Yang[1]

[1]College of Animal Science and Technology, Jilin Agricultural University, Changchun 130118, China.

[2]Key Laboratory of Animal Nutrition and Feed Science of Jilin Province, Changchun 130118, China.

[3]Key Laboratory of Animal Production, Product Quality and Security, Ministry of Education, Changchun 130118, China.

[4]Grassland Research Institute of CAAS, Hohhot 010010, China.

Correspondence and requests for materials should be addressed to H.-M.S. (email: shangmei2000@163.com).

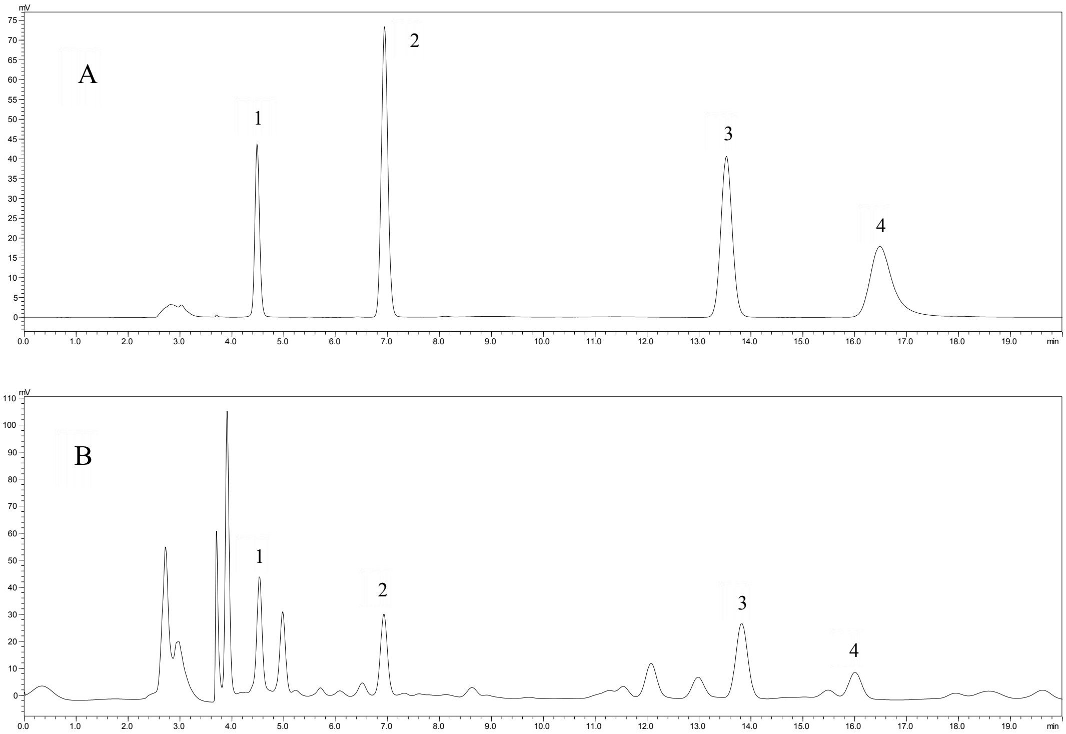

Figure S1. HPLC spectra of phenolic compounds: phenolic compounds reference (A); phenolic compounds of chicory leaves (B) (1, chlorogenic acid; 2, caffeic acid; 3, ferulic acid; 4, Chicoric acid).
